# Pain in cancer. An outcome research project to evaluate the epidemiology, the quality and the effects of pain treatment in cancer patients

**DOI:** 10.1186/1477-7525-4-7

**Published:** 2006-02-02

**Authors:** Giovanni Apolone, Oscar Bertetto, Augusto Caraceni, Oscar Corli, Franco De Conno, Roberto Labianca, Marco Maltoni, Mariaflavia Nicora, Valter Torri, Furio Zucco, the Cancer Pain Outcome Research Study Group

**Affiliations:** 1 Laboratorio di Ricerca Traslazionale e di Outcome, Dipartimento di Oncologia, Istituto di Ricerche Farmacologiche "Mario Negri", Via Eritrea 62, 20157 Milan, Italy; 2 Oncologia Medica, Ospedale Molinette, C.so Bramante 88, 10126 Turin, Italy; 3 Unità Operativa Cure Palliative, Istituto Nazionale dei Tumori, Via Venezian 1, 20123 Milan, Italy; 4 Unità Operativa Cure Palliative, Istituti Clinici di Perfezionamento, P.O. "Vittore Buzzi", Via Castelvetro 32, 20154 Milan, Italy; 5 Oncologia Medica, Ospedali Riuniti di Bergamo, Largo Barozzi 1, 24100 Bergamo, Italy; 6 Hospice Forlimpopoli, ASL Forli Cure Palliative Via Duca D'Aosta 33, 47034 Forlimpopoli (FC), Italy; 7 Direzione Medica, Grunenthal-Formenti, Via Correggio 43, 20149 Milan, Italy; 8 Laboratorio di Ricerca Clinica Oncologica, Dipartimento di Oncologia, Istituto di Ricerche Farmacologiche "Mario Negri", Via Eritrea 62, 20157 Milan, Italy; 9 Hospice Garbagnate, Cure Palliative AO Salvini Viale Forlanini, 121 20024 Garbagnate Milanese (MI), Italy

## Abstract

**Background:**

Management of pain related to advanced or metastatic cancer, although the availability of several pharmacological and non-pharmacological interventions and the existence of well-known guidelines and protocols, is often difficult and inadequate. Evidence of the relative effectiveness of current options for treating cancer pain from comparative randomized studies is scanty.

**Methods:**

In the context of a wider project, a multicenter, open label, prospective Outcome Research study will be launched in Italy in 2006 to investigate the epidemiology of cancer pain and of its treatments, the quality of analgesic-drug therapy and the effectiveness of alternative analgesic strategies in a large, prospective, unselected cohort of cancer patients using the state-of-the art of patient-reported-outcomes. About 100 Italian centers will recruit 2500 patients with advanced/progressive/metastatic cancer with pain (related to the cancer disease) requiring analgesic treatments. Each center is expected to recruit 25 consecutive and eligible patients during the study inception period. Approximately two months will be allowed for subject recruitment and enrollment. Subject evaluation and follow-up will be for 3 months. The effect on outcomes of various therapeutic analgesic options administered by physicians, given the observational approach where patients are not assigned at random to different treatments, will be compared using the propensity score approach, allowing the adjustment for treatment selection bias. Later, after the launch of the observational study and on the basis of results, in specific subsamples of patients and in select centers of the network, a Randomized Controlled Trial will be carried out to formally compare the efficacy of alternative analgesic strategies, with particular emphasis on oral morphine (as comparator) and buprenorphine patch (as experimental arm). Results from the outcome (cohort) and experimental (Randomized Controlled Trial) studies will ensure both the external and internal validity.

## Background

Pain afflicts most cancer patients, mainly in the advanced and metastatic phase of the disease [1]. Recent reviews of published literature suggest that the prevalence of pain in advanced cancer is about 70% with ample variations according to the cancer type and disease stage [2].

Despite the existence of published and well-known guidelines for cancer pain management recommended by the World Health Organization (WHO) and effective treatments are available for 70–90% of cases [3], under treatment is well documented and large proportions of cancer patients (reaching in some evaluations 40%) remain intentionally under-treated [4] for several reasons, often conceptualized in terms of barriers related to health care provider, patient, family, institution and society [5]. The most frequent cause of under-treatment is usually considered misconceptions about opioids [6].

Evidence about pain prevalence, drug utilization and treatments effectiveness in cancer patients are scanty, most deriving from placebo trials, with very few comparative studies in large and well characterized cohorts of cancer patients. Furthermore, it is not available in medical literature any large parallel randomized controlled trial conducted in advanced cancer patients suffering from severe pain (step 3 in the WHO's pain ladder), comparing any alternative opioids to oral morphine [7-10].

The general picture that derives from the analysis of experiences carried out in international settings does apply to Italy also, with some peculiarities: *a*) Opioids consumption is ranked among the lowest in Europe until 2000, with some improvements at least in terms DDD/100,000 people after the introduction of new opiods in the list of reimbursable drugs (transdermal fentanyl and morphine syrup) in 2000 [11]. *b*) Until January 2005, when a new bill was introduced at national level, no WHO-step II analgesic products were available in the list of reimbursable drugs in most of Italian regions, as well as some "strong" WHO-step III opioids, making it difficult for physicians the appropriate prescription of drugs and creating variability in drugs utilization across different Italian regions. *c*) A non appropriate use of transdermal fentanyl can be suspected, as too many patients received it as first option, although recommended only when oral morphine is inadequate [12].

A multidisciplinary Advisory Board of experts in the field of cancer and pain treatments from Industry, Scientific Societies and Patients Associations was convened by the Mario Negri Institute on November 2003 to discuss the quality of pain management in cancer patients in Italy and, eventually, to recommend possible ameliorative educational and research strategies. Member of the Boards highlighted the lack of and the need for new empirical evidence about the epidemiology and the effectiveness of available therapeutic strategies, and called for a prospective program of research aimed at making available to treating physicians, patients and families valid information about pain management in cancer. Two main lines of activities were identified as necessary: information and education activities addressed to physicians, care givers and patients/citizens, and prospective data collection to document patterns of care and outcomes.

Operationally, some working groups met several times during 2004, identified a selected sample of about 100 centers treating cancer patients with advanced/metastatic disease and pain that could be potentially eligible for a prospective study, and planned 3 types of activities to be implemented during the following 3 years (2005–2007): a) information and education: a prototype of educational course for professionals (physicians and nurses) has been assembled and tested on a sample of participants (through a residential course approved by the Italian Committee for Medical Continuing Education with 26 and 33 credits for physicians and nurses, respectively); a critical appraisal of information available in the web (Internet) was also carried out with the preparation and publication of a meta-site that facilitates the use of selected (internet) resources for people interested and/or involved in issues related to cancer pain [13]; b) drug utilization and appropriateness: an evaluation of the volume and quality of prescriptions of analgesic drugs in a large administrative data base in collaboration with local Italian Health Authorities has been started to test the feasibility of the approach in a wider context (regional); c) a prospective, multicenter, nationwide effectiveness study to test the effect of different and alternatives analgesic strategies (such as oral morphine, fentanyl and buprenorphine patches, etc) on patient-reported-outcomes.

The above initiatives are fully described elsewhere [14,15]. In the present paper we provide details about the prospective multicenter study, involving more than 100 Italian centers that has the objective to assess the epidemiology and quality of different and alternatives analgesic strategies (such as oral morphine, fentanyl and buprenorphine patches, etc) and their effect on patient-reported-outcomes. Results are expected by the end of 2006.

### Study rationale

Evidence of the relative effectiveness of current options for treating cancer pain from comparative randomized studies is scanty. This study will investigate the epidemiology of cancer pain and of its treatments, the quality of analgesic-drug therapy and the effect of alternative analgesic strategies in a large, prospective, unselected cohort of cancer patients using the state-of-the art of patient-reported-outcomes. Later, Later, after the launch of the observational study and on the basis of results, in specific subsamples of patients, in select centers, a Randomized Controlled Trial (RCT) will be carried out to formally compare the efficacy of alternative analgesic strategies, with particular emphasis on oral morphine as comparator and buprenorphine patch as experimental arm. Results from the outcome and experimental studies will ensure both the external and internal validity. This kind of approach - a comprehensive cohort design study where all participants are followed up regardless of their randomization status, and outcomes of people who participated in the RCT can be compared with those who participated in the cohort study to assess their similarities and differences - is ideal for trials in which large proportions of eligible individuals are likely to be not entered in the RCT [14,16-18].

### Objectives

#### Primary objective

- To describe the characteristics of a large sample of cancer patients in terms of case mix (patients characteristics), pattern of care (pharmacological and non-pharmacological treatments) and relevant efficacy (analgesic and other patient-reported outcomes (PRO) outcomes such satisfaction, quality of life measures) and safety outcomes (generic, disease and treatments specific).

- To assess the quality of analgesic treatments in terms of congruence between patients' reported level of pain (intensity) and the potency of the prescribed analgesic drug (according to the WHO analgesic ladder) [19].

#### Secondary objectives

The database will be used to evaluate the effects of various therapeutic analgesic options administered by physicians to patients. Given the observational setting, outcomes of subjects who were not assigned at random to different treatments will be compared using the propensity score approach, allowing the adjustment for treatment selection bias [20], as recently done in some large observational population studies [21,22]. The reference group will be those patients treated with oral morphine. The propensity analyses will be carried out:

- To evaluate the effectiveness of treatment regimens on pain intensity and patient-reported quality of life

- To evaluate the effect of treatment regimens on patient-reported satisfaction

- To evaluate the safety profile (adverse and side effects) of alternative treatment regimens

## Methods

### Study design

This study will be designed as a multi-center, open-label, observational non-interventional trial in at least 100 centers, in Italy. Approximately 25 subjects will be enrolled in each center. Subjects will be treated by physicians according to the usual practice (standard of care) and followed over time using standardized methods and forms to make possible a valid and reliable description of case-mix, pattern of care and outcomes. Subjects will be monitored to evaluate the analgesic effects for 28 days (4 weeks) and then with a simplified scheme for further 8 weeks.

### Scheduled study specific assessments will be done as follows (Table 1)

**Table 1:** Schedule of assessments and procedures

Procedure	Study Day								
	Screening baseline Day 1*	Telephone contact 1 Day 2	Telephone contact 2 Day 3	Visit Day 4	Visit Day 7*	Visit Day 14*	Visit Day 21*	Visit Day 28*	Final Visit Week 12 *
Informed consent	x	-	-	-	-	-	-	-	-
Eligibility criteria	x	-	-	-	-	-	-	-	-
Study registration	x	-	-	-	-	-	-	-	-
Medical history	x	-	-	-	-	-	-	-	x
Physical examination	x	-	-	x	x	x	x	x	x
Analgesic consumption	x	-	-	-	x	x	x	x	x
Pain assessment (BPI)	x	x	x	x	x	x	x	x	x
Symptoms and Side effects assessment	x	x	x	x	x	x	x	x	x
Patient global assessment	x	-	-	-	x	x	x	x	x
Physician global assessment	x	-	-		x	x	x	x	x
HRQOL	x	-	-	-	x	x	x	x	x

*mandatory

- Baseline assessment (First visit).

- Telephone contacts 24 and 48 hours after the inclusion (facultative).

- Visit at day 7,14,21,28.

- Final complete assessment at month 3 (12 weeks from inclusion and 8 from last complete evaluation) regarding patients' (analgesic and non-analgesic) outcomes and vital status; a summary of treatments delivered during the period is also planned.

### Subject population

All patients, either new cases or already followed-up at the participating centers, with eligible (advanced or metastatic) solid cancers and with pain (related to cancer disease) requiring any treatment are eligible to be included during the study inception period.

### Study period and number of cases per-center

Each center is expected to recruit 25 consecutive and eligible patients during the study inception period.

Approximately two months will be allowed for subject recruitment and enrollment. Subject evaluation and follow-up will be for 3 months.

### Study conduct and supervision

In order to be able to conduct and monitor the progress of the trial for the patients' safety and according to the World Medical Association Declaration of Helsinki and the European and Italian Good Clinical Practice guidelines, as well as to establish and guide the Group publication policy, several Committees, Boards and Groups have been assembled and organized in a pre-planned Trial Structure and Organization, with explicit guidance for its functioning. Basically, 4 hierarchical bodies will be created: an Advisory Board, a Steering Committee, a Protocol Writing Committee and a Project Management Group (Appendix 1 [see Additional file 1]). If necessary, a Committee for Ancillary Studies (CAS) will be also activated.

### Entry criteria

All patients seen at the participating centers during the study inception period will be screened, and if considered eligible included, registered and monitored over time. After a check of the eligibility and inclusion in the study, the investigator will assign treatments to patients according to the standard practice (usual care). In the case of prescription of WHO step-II and -III drugs, appropriate strategies will be recommended to reach the adequate individual pain control, with particular attention to titration, identification and management of side effects.

### Eligibility (inclusion) criteria

The study will include patients:

1. with diagnostic (histological or cytological) evidence of advanced/progressive/metastatic solid tumor;

2. with pain, any degree of intensity (assessed on a numerical scale from 0 to 10 by asking the patient to rate the *average *intensity of his/her pain over the last 24 hours), related to cancer, requiring analgesic treatment or already on analgesic treatment;

3. with age ≥ 18 years;

4. with a life expectancy of greater than 1 month;

### Exclusion criteria

A subject must not meet any of the following exclusion criteria to be eligible in the study:

1. the subject is less than 18 years of age;

2. the subject is participating in other research projects that conflict or may confound the results of the present study;

3. the subject is unwilling or unable to provide a valid consent for the participation;

4. the subject has some medical conditions, including psychiatric/mental illness, severe senile or Alzheimer' dementia, substance abuse, deemed by the Investigator to be likely to interfere with participation and compliance with the study protocol;

5. the subject cannot guarantee regular follow-up visits for logistic or geographic reasons.

### Study medications (drugs supply) and other medical procedures

Pharmacological and non-pharmacological interventions, prescribed by physicians in the context of their best practice for cases included in the prospective cohort study are provided free as in all centers they are standard care for patients requiring analgesic treatments for pain control, according to each center and investigator policy. In the case a RCT will be activated in the context of the study, drugs compared in the RCT part of the study will be furnished by the Sponsor(s).

All medications must be documented on the CRF.

### Informed consent

Subjects must be able to provide valid written consent, approved by the local Ethics Committee centrally by the study coordinating center.

### Subject enrollment

After consent is obtained, a subject will be enrolled when he/she meets all the inclusion criteria and none of the exclusion criteria. A sequential site-patient number will be assigned.

### Baseline Assessment

After enrollment/inclusion, the following screening assessments will be performed and recorded: a) medical history including past cancer history, b) physical examination, c) recording of medications and recent therapies, including analgesic consumption, d) pain assessment using the Brief Pian Inventory (BPI), e) symptoms and side effects assessment, f) patient and physician global assessment (PGA), g) patient's self-reported health-related quality-of-life (HRQOL).

### Follow-up assessments

According to the flow-chart (Table 1), some evaluations are recommended and others are compulsory. In addition to the first baseline assessment, the following evaluations are mandatory: day 7,14,21,28.

### Final (end-of-study) visit

At week 12 (3 months after the inclusion) a complete re-assessment is required: a) medical history including past cancer history, b) physical examination, c) recording of medications and recent therapies, including analgesic consumption, d) pain assessment using the BPI, e) symptoms and side effects assessment, f) patient and physician global assessment, g) patient's self-reported health-related quality-of-life.

### Pain assessment

Pain will be assessed with the BPI [19,23,24] which will be administered at baseline, at day 7, 14,21,28 and at week 12 (3 months) when patients will attend regular visits at the center or during admission or at home depending on the setting of care (outpatient clinic, hospice, hospital unit, home care).

### Quality of analgesic care assessment

The quality of analgesic treatments will be cross-sectionally assessed in terms of congruence between the patients' reported level of pain (intensity) and the potency of the prescribed analgesic drug (according to the WHO analgesic ladder). Operationally, self-reported pain intensity (from the BPI) and the level of most potent analgesic prescribed (from the CRF) will be combined to assemble a Index of Pain Management (IPM) that ranges from -3 (a patient with severe pain receiving no analgesic drugs) to +3 (a patient receiving morphine or an equivalent drug an reporting no pain) [19]. Data collected longitudinally will be used to test the validity and yield of other dynamic approaches to assess the quality of analgesic care at individual level.

### Quality of life and other relevant patient reported outcomes

Quality of life, satisfaction measures and symptoms/side effects check-lists will be administered to patients when planned during the follow-up [25-27].

Information about all pain medications and number of rescue doses assumed by patients will be also recorded in the CRF.

### Safety issues

All adverse events, serious and non-serious, as well as generic, disease and treatment specific symptoms, encountered during the clinical study will be reported on the appropriate page of the CRF.

### Sample size estimates

According to data available from the literature and to preliminary results from an ongoing observational Italian study [28], we may assume that 100 centers during a inception (recruitment) period of 2 months can include and evaluate about 2.500 eligible cases. Half of them will be already receiving a WHO-step III treatment. Most (60%) of the remaining 1250 will eventually need a WHO-Step III analgesic drug (750) during the longitudinal evaluation period.

Overall, considering the inception and the longitudinal evaluation, the entire duration of the study is estimated around 5 months. At the moment, more than 160 centers accepted to participate and 130 have successfully carried out a pilot phase of the study to test the study feasibility (Appendix 2 [see Additional file 2]).

### Data analysis and statistics

According to the first main objective of the study (i.e., to describe the characteristics of cancer patients in terms of patients characteristics, pharmacological and non-pharmacological treatments and relevant analgesic and other PRO outcomes such as satisfaction, quality of life, generic, disease and treatments specific side effects), all patients enrolled in the study and eligible will be included in the statistical descriptive analyses. To avoid and minimize the impact of missing data, a bias that may be present in the case of using patient-reported measures as principal outcomes/endpoints [29-32], particularly when patients discontinue study treatment, specific procedures to treat missing items will be used, such as the last observation carried forward -LOCF. Data will be analyzed using either parametric and non-parametric statistical tests. When appropriate, for example for the weekly pain ratings scales, scores will be analyzed using repeated analyses of variance to analyze and separate the effects of treatments and time and test the interaction between the two, as well [33].

As to the assessment of the quality of analgesic care, the adequacy of pain management will be assessed firstly using the IPM, an index assembled by grouping with explicit and pre-planned rules patient with different intensity of pain and level of analgesic therapies [19]. Although IPM is not accurate for prescribing drug to an individual, it provides a rough estimate of how pain is treated in the population. According to the literature, each patient will be classified into one of seven mutually exclusive levels of care (from -3 to +3) and appropriate univariate and multivariate analyses will be carried out on the whole sample and on relevant sub-groups to screen predictors of poor analgesic care and to identify patients at greater risk of under-medication. Data collected over time will be used to develop and test new methods to assess quality of analgesic care that may be used at individual (single case) level.

As to the secondary objective of the study (i.e., to evaluate the effects of various therapeutic analgesic options administered by physicians to patients), given the non-randomized design the propensity score approach [20-22] will be used to balance the covariates in the groups and thus reduce the bias produced by the lack of randomization. Briefly, in all 3 comparisons planned by protocol (oral morphine vs fentanyl patch, oral morphine vs buprenorphine patch, fentanyl vs buprenorphine), a comparison specific propensity scores will be estimated. The propensity score method implies a comprehensive collection of information known to be causally associated with the choice of the treatments, then the production of a propensity score (the conditional probability of exposure to a treatment given the observed covariates) using standard statistical models such as logistic regressions to predict the treatment (for example, the choice of morphine in eligible patients), eventually the comparison of outcomes (for example, pian intensity) by matching or stratifing patients according to the value of the propensity scores.

### Study endpoints

The Study will collect, describe and assess a vast array of outcomes measures related to pain and to its impact on patients' life. Some of these measures will be collected and reported by physicians and other directly by patients.

### Pain related outcomes

The predefined primary outcome measurement is the pain intensity over the first weeks, assessed with four questions from the BPI, that will be used either individually or after the computation of their mean, as indicated by developers [19,23,24]. The relevant endpoint is the reduction in worst and *average pain score *of ≥ 2 points (pain intensity difference, PID, >/= 2) at week 4 (day 28 from inclusion) over baseline.

### Other endpoints

Escape/Rescue pain medications: counts of rescue/escape medications taken to control pain from inclusion to week 4 (day 28 from inclusion).

Adequacy of analgesic-drug therapy: Patient Management index (PMI), categorized in a binary variable according to negative (indicating inadequate analgesic care) and positive (conservative indicator of acceptable treatment) levels.

Other patient-reported endpoints:HRQOL, Satisfaction with (drugs) treatment, rate and severity of symptoms, adverse and side effects.

### Ethical approval and publication policy

Before entering patients into the cohort study, clinicians/investigators must ensure that the Protocol has received clearance from the Local Research Ethics Committee (L-REC). The patients' consent to participate in the studies should be obtained after a full explanation has been given. Particular attention should be given to explaining the purposes, manner of the management and use of the patients' data from questionnaires and diaries. The right of the patient to withdraw from the studies in any time, without giving reasons, must be respected.

The responsibility for drafting the manuscripts related to the analyses described in this protocol will lie with the WPC whose members will be the primary Authors. At least one representative from high-recruiting centers will be included among the co-authors, and all participants will be acknowledged in the Appendices. All authors should review and approve the manuscript before submission. At least two papers are expected: the first reporting on the results of the "epidemiological" part of the study, including a description of patients' characteristics in terms of baseline socio-demographic and clinical variables, pattern of care and main outcomes, and a discussion of the feasibility of this kind of approaches in the specific setting, the second about the effects (effectiveness) of treatments on patients (and physicians) reported outcomes.

## Supplementary Material

Additional File 1 Appendix 1. Advisory Board, Steering Committee, Protocol Writing Committee and Project Management Group Click here for file

Additional File 2 Appendix 2. Members of the Cancer Pain Outcome Research Study Group Click here for file
