# Supplementary material for: Pain in cancer. An outcome research project to evaluate the epidemiology, the quality and the effects of pain treatment in cancer patients
Source: Health Qual Life Outcomes. 2006 Feb 2;4:7. doi: 10.1186/1477-7525-4-7 (PMC1402259; doi:10.1186/1477-7525-4-7)
Supplement: Additional File 1 — Appendix 1. Advisory Board, Steering Committee, Protocol Writing Committee and Project Management Group [file 1477-7525-4-7-S1.doc]

**Appendix 1. Advisory Board, Steering Committee, Protocol Writing Committee and Project Management Group**

**Advisory Board**

*Livia Pomodoro, Vittorio Ventafridda, Umberto Veronesi*

**Steering Committee**

*Dino Amadori, Giovanni Apolone, Edoardo Arcuri, Oscar Bertetto, Angelo Bianco,*

*Loredana Carbone Floriani, Oscar Corli, Alberto Costa, Franco De Conno,*

*Andrea Galanti, Aron Goldhirsch, Stefano Inglese, Roberto Labianca,*

*Sebastiano Mercadante, Paola Mosconi, Francesco Nicosia, Marcello Tamburini,*

*Furio Zucco*

**Writing Protocol Committee**

*Giovanni Apolone, Oscar Bertetto, Augusto Caraceni, Oscar Corli, Franco De Conno*

*Roberto Labianca, Marco Maltoni, Mariaflavia Nicora, Valter Torri, Furio Zucco*

**Project Management Group**

*Giovan*ni *Apolone* (Principal Investigator),

*Valter Torri* (Statistician), *Paola Mosconi* (Patient-Reported Outcomes),

*Simone Mangano* (Informatics), *Emanuele Negri* (Data Manager),

*Simona Stupia* (Secretarial Support)

*Giulio Isola* (Liaisons with Sponsors)
