# Supplementary material for: Pain in cancer. An outcome research project to evaluate the epidemiology, the quality and the effects of pain treatment in cancer patients
Source: Health Qual Life Outcomes. 2006 Feb 2;4:7. doi: 10.1186/1477-7525-4-7 (PMC1402259; doi:10.1186/1477-7525-4-7)
Supplement: Additional File 2 — Appendix 2. Members of the Cancer Pain Outcome Research Study Group [file 1477-7525-4-7-S2.doc]

# Appendix 2. Members of the Cancer Pain Outcome Research Study Group

|  | ***Abruzzo*** | Ospedale Civile S. Nicola e Filippo  Oncologia Medica  Via G. Di Vittorio - 67051 Avezzano (AQ) Responsabile dr. Recchia Francesco [frecchia1946@libero.it](mailto:frecchia1946@libero.it)  dr. Saggio Gaetano |
| --- | --- | --- |
|  |  | Policlinico di Chieti Università D’Annunzio Oncologia Medica Loc. Madonna delle Piane - 66100 Chieti Responsabile dr. Iacobelli Stefano [iacobell@unich.it](mailto:iacobell@unich.it)  dr. De Tursi Michele |
|  |  | Azienda Ospedaliera Val Vibrata ASL 6  Oncologia Medica  Contrada alla Salara - 64027 Sant’Omero (Teramo)  *Responsabile dr. Pasqualoni Esther*  [panamedeo@libero.it](mailto:panamedeo@libero.it)  dr. Pancotti Amedeo |
|  | ***Basilicata*** | Azienda Ospedaliera San Carlo  Oncologia e Medica Via P. Petrone - 85100 Potenza  *Responsabile dr. Manzione Luigi*  [luigimanzione@libero.it](mailto:luigimanzione@libero.it)  dr. Romano Rosangela |
|  |  | Ospedale Oncologico Regionale  Oncologia Medica Strada Prov..8 Vulture - 85028 Rionero Vulture (Pz) Responsabile dr. Di Renzo Nicola [ndirenzo@crob.it](mailto:ndirenzo@crob.it)  dr. Ardito Raffaele |
|  |  | Azienda Ospedaliera S. Maria delle Grazie  Oncologia Medica  Contr. Cattedra Ambulante - 75100 Matera  *Responsabile dr. Susi Marina*  [segreteriadanzi@hotmail.it](mailto:segreteriadanzi@hotmail.it)  dr. Brucoli Immacolata |
|  | ***Calabria*** | Azienda Ospedaliera Pugliese-Ciaccio  Oncologia Medica Via Pio X - 88100 Catanzaro  *Responsabile dr. Molica Stefano*  [oncocz@libero.it](mailto:oncocz@libero.it)  dr. Misuraca Demetrio |
|  |  | Azienda Ospedaliera Mariano Santo  Oncologia Medica contrada Muoio Piccolo - 87100 Cosenza  *Responsabile dr. Palazzo*  [salvatore.palazzo@tiscalinet.it](mailto:salvatore.palazzo@tiscalinet.it)  dr. Amato Francesco |
|  |  | Osp Riuniti Bianchi Morelli  Oncologia Medica Via Melacrino - 89100 Reggio Calabria  *Responsabile dr. Nardi Mario*  [onc.nardi@virgilio.it](mailto:onc.nardi@virgilio.it)  dr. Giuffrè Caterina |

|  | ***Campania*** | AO Policlinico Università Federico II Oncologia Medica II  Via Pansini, 5 - 80131 Napoli  *Responsabile dr. Bianco Angelo; dr. De Placido Sabino*  [bianco@unina.it](mailto:bianco@unina.it)  dr. Matano Elide |
| --- | --- | --- |
|  |  | Policlinico Federico II  Terapia del Dolore e Cure Palliative  Via Pansini, 5 - 80131 Napoli  *Responsabile dr. Palomba Rosa*  [giovannini47@aliceposta.it](mailto:giovannini47@aliceposta.it)  dr. Di Maria Carmen |
|  |  | Istituto Naz.le dei Tumori Fondazione Pascale  Terapia Antalgica e Rianimazione  Via M. Semmola - 80131 Napoli  *Responsabile dr. Cuomo Arturo*  [arturocuomo@libero.it](mailto:arturocuomo@libero.it)  dr. Duraccio Daniela |
|  |  | Istituto Naz.le dei Tumori Fondazione Pascale  Oncologia Medica A Via M. Semmola - 80131 Napoli Responsabile dr. Comella Giuseppe [giuseppe.comella@libero.it](mailto:giuseppe.comella@libero.it)  dr. Sandomenico Claudia |
|  |  | Istituto Naz.le dei Tumori Fondazione Pascale  Oncologia Medica B Via M. Semmola - 80131 Napoli  *Responsabile dr. Iaffaioli Rosario*  [eiaffaioli@libero.it](mailto:eiaffaioli@libero.it)  dr. Facchini Gaetano |
|  |  | Istituto Naz.le dei Tumori Fondazione Pascale  Oncologia Medica C Via M. Semmola - 80131 Napoli  *Responsabile dr. De Matteis Andrea*  [dematteisandrea@libero.it](mailto:dematteisandrea@libero.it)  dr. Rossi Emanuela |
|  |  | Istituto Naz.le dei Tumori Fondazione Pascale  Sperimentazioni Cliniche  Via M. Semmola - 80131 Napoli Responsabile dr. Perrone Francesco [fr.perrone@agora.it](mailto:dimaiomax@libero.it)  dr. Di Maio Massimo |
|  |  | Azienda Ospedaliera Cardarelli  Oncologia Via Cardarelli, 9 - 80131 Napoli  *Responsabile dr. Chiurazzi Bruno*  [bchiura@tin.it](mailto:bchiura@tin.it)  dr. Carteni Giacomo |
|  |  | Azienda Ospedaliera Monaldi  II U.O. Pneumologia Oncologica  Via Leonardo Bianchi - 80131 Napoli  *Responsabile dr. Brancaccio Luigi*  [luigi.brancaccio@ospedalemonaldi.it](mailto:luigi.brancaccio@ospedalemonaldi.it)  dr. Brunello Valentino |
|  |  | Azienda Ospedaliera Monaldi  I Pneumo Oncologia Via Leonardo Bianchi - 80131 Napoli  *Responsabile dr. Crispino Carlo*  [cvd.crispino@libero.it](mailto:cvd.crispino@libero.it)  dr. Chianca Pio |
|  |  | Azienda Ospedaliera San Gennaro  Oncologia Medica Via S.Gennaro dei Poveri 25 - 80135 Napoli  *Responsabile dr. De Sangro Carlo; dr. Leopardi Luigi*  [carlo.desangro@tin.it](mailto:carlo.desangro@tin.it)  dr. Maiorino Luigi |
|  |  | Osp.San Giuliano ASL NA2  Oncologia Medica  Via G.B. Basile - 80014 S. Giuliano (Napoli)  *Responsabile dr. Incoronato Pasquale*  [pasquale.incoronato@tin.it](mailto:pasquale.incoronato@tin.it)  dr. Di Lanno Maria |
|  |  | Osp S. Giovanni di Dio ASL3  Oncologia Via Pirozzi - 80027 Frattamaggiore (Na)  *Responsabile dr. Del Prete Salvatore*  [saldelprete@yahoo.it](mailto:saldelprete@yahoo.it)  dr. Faiola Vincenzo |
|  |  | Osp Santa Maria della Pietà  Oncologia Medica  Via Seminario - 80035 Nola (Na)  *Responsabile dr. Bencivenga Giuseppina*  [pinabenci@libero.it](mailto:pinabenci@libero.it)  dr. Tufano Maria Luisa |
|  |  | Ospedale Apicella  Oncologia Medica  Via Purgatorio – 80040 Pollena Trocchia (Na)  *Responsabile dr. Tufano Maria Luisa*  [anagrafe@comunesantanastasia.it](mailto:anagrafe@comunesantanastasia.it)  dr. Bencivenga Giuseppina |
|  |  | Osp Sacro Cuore Fatebenefratelli  Terapie Antalgiche  V.le Principe di Napoli 14/a - 82100 Benevento Responsabile: dr. Di Gregorio Renata [renatadigregorio@libero.it](mailto:renatadigregorio@libero.it)  dr. Bardari Giovanna |
|  |  | AO Giovanni da Procida ASL 2 Salerno  Oncologia  Via S. Calenda - 84100 Salerno  *Responsabile dr. De Cataldis Giuseppe*  [gidecataldis@tiscali.it](mailto:gidecataldis@tiscali.it)  dr. Carnicelli Pietro |
|  |  | Azienda Ospedaliera Umberto I ASL Salerno 1  Oncoematologia via San Francesco - 84014 Nocera Inferiore (Sa)  *Responsabile dr. Libroia Anna*  [doctannalibroia@virgilio.it](mailto:doctannalibroia@virgilio.it)  dr. Fabbrocini Antonietta |
|  |  | Assistenza Domiciliare Oncologica  Dip.to Oncologico  Via Atzori, 64/7 - 84014 Nocera Inferiore (Sa) Responsabile dr. Dello Ioio Concetta [c.delloioio@lapiramide.org](mailto:c.delloioio@lapiramide.org)  dr. Coppola Luciano |
|  |  | ASL Salerno 2 Distretto 103  Terapia del Dolore  Via F.lli Adinolfi - 84025 Eboli (Sa)  *Responsabile dr. De Martino Armando*  [armandodemartino@inwind.it](mailto:armandodemartino@inwind.it)  dr. Maiorano Cosimo |
|  |  | A.O. San Sebastiano  Terapia del dolore e Cure Palliative  Via Palasciano - 81100 Caserta  *Responsabile dr. Zeppetella Gianluigi*  [terapiadolore@ospedale.caserta.it](mailto:terapiadolore@ospedale.caserta.it)  dr. Ievoli Luigi |
|  |  | Ospedale Moscati  Oncologia Medica  Via Ligniti – 83024 Monteforte Irpino (Av) Responsabile dr. Gridelli Cesare [oncologia-avellino@libero.it](mailto:oncologia-avellino@libero.it)  dr. Ferrara Carmine |
|  | ***Emilia Romagna*** | Azienda Ospedaliera Bellaria  Oncologia Medica Via Altura, 3 - 40139 Bologna  *Responsabile dr. Maestri Antonio*  [antonio.maestri@ausl.bo.it](mailto:antonio.maestri@ausl.bo.it)  dr. Calandra Cesare |
|  |  | Policlinico. S.Orsola Malpighi Oncologia Medica  Via Albertoni, 15 - 40138 Bologna  *Responsabile dr. Martoni Andrea*  [martoni@aosp.bo.it](mailto:martoni@aosp.bo.it)  dr. Cricca Antonia |
|  |  | Hospice Chiantore Seragnoli  Via Marconi 43 - 40010 Bentivoglio (Bo)  *Responsabile dr. Valenti Danila*  [danila.valenti@hospiceseragnoli.it](mailto:danila.valenti@hospiceseragnoli.it)  dr. Negretti Carla |
|  |  | Centro Oncologico Azienda Ospedaliera Modena  Hospice Modena Via del Pozzo, 71 - 41100 Modena  *Responsabile dr. Dini Daniele*  [dini.d@policlinico.mo.it](mailto:dini.d@policlinico.mo.it)  dr. Piccinini Lino |
|  |  | Azienda Ospedaliera Ramazzini  Medicina Oncologica  Via Molinari, 2 - 41012 Carpi (Mo)  *Responsabile dr. Bandieri Elena*  [e.bandieri@ausl.mo.it](mailto:e.bandieri@ausl.mo.it)  dr. Cagossi Katia |
|  |  | Azienda Ospedaliera Guglielmo da Saliceto  Oncologia  Via Cantone del Cristo 40 - 29100 Piacenza  *Responsabile dr. Cavanna Luigi*  [l.cavanna@ausl.pc.it](mailto:l.cavanna@ausl.pc.it)  dr. Bertè Raffaella |
|  |  | Ospedale Maggiore  Oncologia Medica Via Gramsci, 14 - 43100 Parma Responsabile dr. Vasini Giovanna [gvasini@ao.pr.it](mailto:gvasini@ao.pr.it)  dr. Spiritelli Elena |
|  |  | Presidio Ospedaliero Fidenza  Cure Palliative Via Don E. Tincati, 5 Loc. Vaio- 43036 Fidenza (Pr) *Responsabile dr. Ghisoni Francesco*  [fghisoni@ausl.pr.it](mailto:fghisoni@ausl.pr.it)  dr. Manelli Daniele |
|  |  | Azienda Ospedaliera Santa Maria Nuova  Oncologia V.le Risorgimento, 80 - 42100 Reggio Emilia  *Responsabile dr. Rondini Ermanno*  [rondini.ermanno@asmn.re.it](mailto:boni.corrado@asmn.re.it)  dr. Linarello Pasquale |
|  |  | Hospice Casa Madonna dell’Uliveto  Oncologia  Via Oliveto 37 - 42020 Montericco di Albinea (Re)  *Responsabile dr. Manni Antonio*  [info@madonna-uliveto.org](mailto:info@madonna-uliveto.org)  dr. Piacentini Mariangela |
|  |  | Ospedale Civile “Magati"  DH Oncologico  Via Martiri della libertà 8 - 42019 Scandiano (Re) Responsabile dr. Cottafavi Luca [cottafavil@ausl.re.it](mailto:cottafavil@ausl.re.it)  dr. Manenti Anna Lucia |
|  |  | Ospedale San Colomba – ASL Cesena  Oncologia  Corso Perticari 117 - 47039 Savignano Sul Rubiconde (FC) Responsabile dr. Turci Paola [pturci@ausl-cesena.emr.it](mailto:pturci@ausl-cesena.emr.it)  dr. Pittureri Cristina |
|  |  | Hospice di Forlimpopoli ASL Forlì  Cure Palliative  Via Duca d’Aosta, 33 47034 Forlimpopoli (FC)  *Responsabile dr. Maltoni Marco* [malto.ma@tin.it](mailto:malto.ma@tin.it)  dr. Fabbri Laura |
|  |  | Ospedale degli Infermi Oncologia via Settembrini, 2 - 47900 Rimini Responsabile dr. Tassinari Davide [dtassinari@rimini.com](mailto:dtassinari@rimini.com)  dr. Poggi Barbara |
|  |  | Azienda Ospedaliera Lugo di Ravenna  Oncologia Medica  Viale Dante, 10 - 48022 Lugo di Ravenna  *Responsabile dr. Montanari Luigi*  [lu.hoonco@ausl.ra.it](mailto:lu.hoonco@ausl.ra.it)  dr. Carrozza Francesco; dr. Montanari Marco |
|  | ***Friuli Venezia Giulia*** | Ospedale di Cattinara Ambulatorio di Terapia Antalgica Via Pietà, 19 - 34100 Trieste  *Responsabile dr. Serra Licia*  [liciaserra@libero.it](mailto:giorgio.mustacchi@ass1.sanita.fvg.it)  dr. Foladore Silvia |
|  |  | Ospedale San Polo ASS 2 Isontino  Oncologia  Via Galvani, 1 - 34074 Monfalcone (Go) Responsabile dr. Frigo Anna Luisa [annalfri@tin.it](mailto:annalfri@tin.it)  dr. Recchia Leonardo |
|  | ***Lazio*** | Policlinico Universitario Gemelli  Radioterapia Largo Gemelli, 8 - 00168 Roma Responsabile dr. Valentini Vincenzo [vvalentini@rm.unicatt.it](mailto:vvalentini@rm.unicatt.it) |
|  |  | Policlinico Umberto I  DH Oncologico Viale Regina Elena 324 - 00161 Roma  *Responsabile dr. Cortesi Enrico*  [enrico.cortesi@uniroma1.it](mailto:enrico.cortesi@uniroma1.it)  dr. Mazzoli Marta |
|  |  | Policlinico Umberto I  Oncologia A  Viale Regina Elena 324 - 00161 Roma  *Responsabile dr. Di Seri Marisa*  [marisa.diseri@uniroma1.it](mailto:marisa.diseri@uniroma1.it)  dr. Proietti Emanuela |
|  |  | Policlinico Umberto I  Terapia del Dolore  Viale del Policlinico 155 - 00161 Roma Responsabile dr. Reale Carlo [carloreale@uniroma1.it](mailto:carloreale@uniroma1.it)  dr. Luzi Marta |
|  |  | Ospedale Regina Elena  Terapia del Dolore Polo Oncologico  Via Elio Chianesi, 53 - 00128 Roma Responsabile dr. Arcuri Edoardo [arcuri@ifo.it](mailto:arcuri@ifo.it)  dr. Tirelli Walter; dr. Di Emidio Loriana |
|  |  | Ospedale Regina Elena  Oncologia C Via Elio Chianesi, 53 - 00128 Roma Responsabile dr. Terzoli Edmondo [terzoli@ifo.it](mailto:terzoli@ifo.it)  dr. Bria Emilio |
|  |  | Policlinico Università Tor Vergata  Oncologia Medica V.le degli Ammiragli, 46 - 00136 Roma  *Responsabile dr. Roselli Mario*  [mario.roselli@ptvonline.it](mailto:mario.roselli@ptvonline.it)  dr. Del Monte Girolamo |
|  |  | Azienda Ospedaliera Sandro Pertini  Oncologia  Via dei Monti Tiburtini 385 - 00157 Roma  *Responsabile dr. Cammilluzzi Eugenio*  [eugenio.cammilluzzi@aslromab.it](mailto:eugenio.cammilluzzi@aslromab.it)  dr. Aversa Alessandro; dr. Gilberti Sara |
|  |  | Azienda Ospedaliera Fatebenefratelli  Oncologia Medica San Giovanni Calabita - Isola Tiberina - 00186 Roma *Responsabile dr. Breda Enrico*  [vzagone@tin.it](mailto:vagone@tin.it)  dr. Zagonel Vittorina |
|  |  | Ospedale San Giacomo  DH Oncologico  Via Canova, 29 – 00186 Roma Responsabile dr. Temperilli Luigi [gezampa@libero.it](mailto:gezampa@libero.it)  dr. Zampa Germano |
|  |  | Università Campus Bio Medico  Oncologia Medica Via Longoni, 83 - 00173 Roma  *Responsabile dr. Tonini Giuseppe*  [g.tonini@unicampus.it](mailto:g.tonini@unicampus.it)  dr. Vincenzi Bruno; dr Santini Daniele |
|  |  | Ospedale Israelitico  Oncologia Medica  Via Fulda, 14 - 00148 Roma  *Responsabile dr. Madaio Raffaele*  [rmadaio@tiscali.it](mailto:rmadaio@tiscali.it)  dr. Gallà Domenico |
|  |  | Ospedale Belcolle ASL Viterbo  Divisione Oncologia Strada Sammartinese - 01100 Viterbo Responsabile dr. Pollera Camillo [cfpollera@oncologiaviterbo.it](mailto:cfpollera@oncologiaviterbo.it)  dr. Nelli Fabrizio [f_nelli@hotmail.com](mailto:f_nelli@hotmail.com) |
|  |  | Azienda Ospedaliera S. Maria Goretti  Oncoematologia Via Canova, 2 - 04100 Latina  *Responsabile dr. Di Palma Teresa*  [teresa_dipalma@yahoo.it](mailto:teresa_dipalma@yahoo.it)  dr. Sciacca Venerina |
|  |  | Centro Oncologico E. Conti, ASL Latina  Onc. Med. Ospedale "Luigi Di Liegro"  Salita Cappuccini - 04024 Gaeta (Lt) Responsabile dr. Veltri Enzo [aslgae.oncolgaeta@tiscali.it](mailto:aslgae.oncolgaeta@tiscali.it)  dr. Cardillo Franca |
|  |  | Azienda Ospedaliera SS.Trinità  Oncologia Medica  Località S. Marciano - 03039 Sora (Fr)  *Responsabile dr. Gamucci Teresa*  [oncologia.frosinone@libero.it](mailto:oncologia.frosinone@libero.it)  dr. Narducci Filomena |
|  | ***Liguria*** | Azienda Ospedaliera San Martino DIMI Terapia del Dolore Pad. 7 Largo R. Benzi, 10 - 16132 Genova  *Responsabile dr. Roy Maria Teresa*  [mariateresa.roy@hsanmartino.liguria.it](mailto:mariateresa.roy@hsanmartino.liguria.it)  dr. Pilastri Paola |
|  |  | ASL 3 Dip.to A. Anzani  Cure Palliative  Via Bonghi, 6 - 16162 Genova  *Responsabile dr. Fusco Flavio*  [flavio.fusco@asl3.liguria.it](mailto:flavio.fusco@asl3.liguria.it)  dr. Silvestro Silvana |
|  |  | Associazione Gigi Ghirotti  Hospice Piazza ex Ospedale Pastorino, 1 - 16162 Genova  *Responsabile dr. Henriquet Franco*  [franco.henri@libero.it](mailto:franco.henri@libero.it)  dr. Davoodi Iraj |
|  |  | Azienda Ospedaliera Galliera  Oncologia Medica e Preventiva Via delle Cappuccine, 14 - 16128 Genova  *Responsabile dr. Caroti Cinzia*  [cinzia.caroti@galliera.it](mailto:cinzia.caroti@galliera.it)  dr. D’Amico Mauro |
|  |  | Istituto Naz. Ricerca sul Cancro  Divisione Terapia del Dolore L.go Rosanna Benzi, 10 - 16132 Genova  *Responsabile dr. Dini Dario*  [dario.dini@istge.it](mailto:dario.dini@istge.it)  dr. Luzzani Massimo |
|  |  | ASL 5 Spezzino Osp Felettino  Terapia del Dolore e Cure Palliative  Via 24 Maggio 139 - 19124 La Spezia  *Responsabile dr. Di Alesio Lorenzo*  [l.dialesio@libero.it](mailto:l.dialesio@libero.it)  dr. Martinetti Mario |
|  | ***Lombardia*** | Fondazione Salvatore Maugeri  Oncologia Medica II Via Ferrata, 8 - 27100 Pavia  *Responsabile dr. Bernardo Giovanni*  [gbernardo@fsm.it](mailto:gbernardo@fsm.it)  dr. Delmonte Angelo |
|  |  | Fondazione Salvatore Maugeri  Medicina del Dolore Via Ferrata, 8 - 27100 Pavia  *Responsabile dr.Paulin Livio*  [cbonezzi@fsm.it](mailto:cbonezzi@fsm.it)  dr. Barbieri Massimo |
|  |  | Fondazione Salvatore Maugeri  Terapie del Dolore e Cure Palliative Via Boezio, 28 - 27100 Pavia  *Responsabile dr. Miotti Danilo*  [dmiotti@fsm.it](mailto:dmiotti@fsm.it)  dr. Bonetti Giovanna |
|  |  | Fondazione Salvatore Maugeri IRCCS Pavia  Unità Cure Palliative Via Ferrata, 8 - 27100 Pavia  *Responsabile dr. Cuomo Annamaria*  [ppreti@fsm.it](mailto:ppreti@fsm.it)  dr. Preti Pietro |
|  |  | Fondazione Salvatore Maugeri AO San Martino  Cure Palliative  Viale dei Mille, 23 - 27035 Mede (Pv)  *Responsabile dr.Ferrari Pietro*  [pferrari@fsm..it](mailto:pferrari@fsm..it)  dr. Preti Pietro |
|  |  | Fondazione Salvatore Maugeri  Riabilitazione Oncologica Via Ferrata, 8 - 27100 Pavia  *Responsabile dr. Strada Maria Rosa*  [mstrada@fsm.it](mailto:mstrada@fsm.it)  dr. Frascaroli Mara |
|  |  | Fondazione Salvatore Maugeri  Oncologia Medica I Via Ferrata, 8 - 27100 Pavia  *Responsabile dr. Pavesi Lorenzo*  [lpavesi@fsm.it](mailto:lpavesi@fsm.it)  dr. Ponchio Luisa |
|  |  | IRCCS Policlinico S.Matteo  Oncologia Medica  Viale Golgi, 19 - 27100 Pavia Responsabile dr. Allegri Massimo [c.porta@smatteo.pv.it](mailto:c.porta@smatteo.pv.it)  dr. Porta Camillo |
|  |  | Istituto Europeo di Oncologia  Chirurgia Generale  Via Ripamonti, 435 - 20141 Milano  *Responsabile dr.Andreoni Bruno*  [bruno.andreoni@ieo.it](mailto:bruno.andreoni@ieo.it) |
|  |  | Istituto Europeo di Oncologia Cure Palliative  Via Ripamonti 435 - 20141 Milano Responsabile dr. Sbanotto Alberto [alberto.sbanotto@ieo.it](mailto:alberto.sbanotto@ieo.it) |
|  |  | Pio Albergo Trivulzio  Hospice  Via Trivulzio, 15 - 20146 Milano  *Responsabile dr. Monti Massimo*  [hospice@pioalbergotrivulzio.it](mailto:hospice@pioalbergotrivulzio.it)  dr. Castellani Lucia |
|  |  | Osp Ca’ Granda Niguarda  Oncologia Medica Falk P.za Ospedale Maggiore, 3 - 20162 Milano  *Responsabile dr. Siena Salvatore*  [oncologia@ospedaleniguarda.it](mailto:oncologia@ospedaleniguarda.it)  dr. Landonio Giuseppe |
|  |  | Osp San Carlo Borromeo  Divisione Oncologia Via Pio II, 2 - 20153 Milano  *Responsabile dr. Vinci Maria*  [seg.oncologia@sancarlo.mi.it](mailto:seg.oncologia@sancarlo.mi.it)  dr. Masseroni Sara |
|  |  | A.O. Luigi Sacco  U.O. di Oncologia  Via G.B. Grassi, 74  20157 Milano  *Responsabile dr. Gabriella Esani*  [oncologiasacco@libero.it](mailto:oncologiasacco@libero.it)  dr. Maria Eugenia Damiani |
|  |  | AO San Paolo  Cure Palliative e Oncologia Medica Via Di Rudinì, 8 - 20142 Milano  *Responsabile dr. Piva Laura*  [laura.piva@ao-sanpaolo.it](mailto:laura.piva@ao-sanpaolo.it) |
|  |  | Casa di Cura Igea  Via Marcona, 69 - 20129 Milano  *Responsabile dr. Pancera Gianfranco*  [gpancera@hotmail.com](mailto:gpancera@hotmail.com)  dr. Garassino Marina |
|  |  | Istituto dei Tumori  Cure Palliative  Via Venezian, 1 - 20133 Milano  *Responsabile dr. De Conno Franco*  [franco.deconno@istitutotumori.mi.it](mailto:franco.deconno@istitutotumori.mi.it)  dr. Caraceni Augusto |
|  |  | Ospedale Civile di Legnano  Oncologia Medica  Via Candiani, 2 - 20025 Legnano  *Responsabile dr. Fava Sergio*  [laelena75@hotmail.com](mailto:laelena75@hotmail.com)  dr. Collovà Elena |
|  |  | Ospedale Civile di Legnano  Terapia del Dolore e Cure palliative  Via Candiani, 2 - 20025 Legnano  *Responsabile dr. Ivanoe Pellerin*  [ivanoe.pellerin@ao-legnano.it](mailto:ivanoe.pellerin@ao-legnano.it) |
|  |  | AO S.Gerardo Osp Bassini  Cure Palliative e Terapia del Dolore  Via Gorky, 50 - 20092 Cinisello Balsamo (Mi)  *Responsabile dr. Speranza Raffaella*  [rafpallio@yahoo.it](mailto:rafpallio@yahoo.it)  dr. De Salve Antimo |
|  |  | Osp. di Circolo Serbelloni  Polo Oncologico  Via Bellini, 5 - 20064 Gorgonzola (Mi)  *Responsabile dr. Isa Luciano*  [luciano.isa@tiscali.it](mailto:luciano.isa@tiscali.it)  dr. Venezia Raffaele |
|  |  | Multimedia  Oncologia Medica  Via Milanese, 300 - 20099 Sesto San Giovanni (Mi)  *Responsabile dr.Gottardi Ornella*  [ornella.gottardi@multimedica.it](mailto:ornella.gottardi@multimedica.it)  dr. Scanzi Francesco |
|  |  | AO San Gerardo  Cure Palliative e Terapia del Dolore  Via Donizetti, 106 - 20052 Monza (Mi)  *Responsabile dr. Castagnini Guia*  [guiacastagnini@virgilio.it](mailto:guiacastagnini@virgilio.it)  dr. Speranza Raffaella |
|  |  | Fondazione Don Gnocchi  Hospice S.M.delle Grazie Via Montecassino, 8 - 20052 Monza (Mi)  *Responsabile dr. Cacioppo Carlo*  [ccacioppo@dongnocchi.it](mailto:ccacioppo@dongnocchi.it)  dr. Scanzi Francesco |
|  |  | Hospice Garbagnate  Cure Palliative AO Salvini viale Forlanini, 121 - 20024 Garbagnate Milanese (Mi)  *Responsabile dr. Zucco Furio*  [rianimazioneg@aogarbagnate.lombardia.it](mailto:rianimazioneg@aogarbagnate.lombardia.it)  dr. Rusconi Maria Grazia |
|  |  | AO Provincia di Lodi  Terapia del Dolore  Viale Savoia, 4 - 26900 Lodi Responsabile dr. Furiosi Domenico [furiodome@hotmail.com](mailto:furiodome@hotmail.com) |
|  |  | A.O. Istituti Ospitalieri di Cremona  Breast Unit  Viale Concordia, 1 - 26100 Cremona Responsabile dr. Bottini Alberto [alberto.bottini@email.it](mailto:alberto.bottini@email.it)  dr. Bonardi Simone |
|  |  | A.O. Istituti Ospitalieri di Cremona  Oncologia Medica  Viale Concordia, 1 - 26100 Cremona Responsabile dr. Passalacqua Roberto [passalacqua.aioc@e-cremona.it](mailto:alberto.bottini@email.it)  dr. Negri Federica; dr. Lazzarelli Silvia |
|  |  | A.O. Istituti Ospitalieri di Cremona  Cure Palliative  Largo Priori, 1 – 26100 Cremona Responsabile dr. Giannunzio Donatella [hosp.aioc@e-cremona.it](mailto:hosp.aioc@e-cremona.it)  dr. De Felice Francesca |
|  |  | Ospedale Maggiore di Crema  Medicina  Largo Dossena – 24016 Crema (Cr)  *Responsabile dr. Bobbio Pallavicini Enrico*  [e.bobbio@hcrema.it](mailto:e.bobbio@hcrema.it)  dr. Bianchessi Chiara Rita |
|  |  | Ospedali Riuniti  Cure Palliative Largo Barozzi, 1 - 24124 Bergamo  *Responsabile dr. Liguori Simeone*  [tdhospice@ospedaliriuniti.bergamo.it](mailto:tdhospice@ospedaliriuniti.bergamo.it)  dr. Soloni Chiara |
|  |  | Ospedali Riuniti  Oncologia Medica Largo Barozzi, 1 - 24124 Bergamo  *Responsabile dr. Moro Cecilia*  [segroncologia@ospedaliriuniti.bergamo.it](mailto:segroncologia@ospedaliriuniti.bergamo.it)  dr. Berardi Eliana |
|  |  | Hospice Domus Salutis Cure Palliative via Lazzaretto, 3 - 25123 Brescia  *Responsabile dr. Zaninetta Giovanni*  [zaninetta.domus@guests.onion.it](mailto:zaninetta.domus@guests.onion.it)  dr. Fasser Nicola |
|  |  | Azienda Ospedaliera Valduce  Oncologia  Via Dante, 11 - 22100 Como  *Responsabile dr. Duro Maria*  [oncologia@valduce.it](mailto:oncologia@valduce.it)  dr. Frigerio Guido |
|  |  | Azienda Ospedaliera Sant’Anna  Terapia del Dolore Via Napoleona, 60 - 22100 Como Responsabile dr. Cerutti Gigliola [*rianim1@hsacomo.org*](mailto:rianim1@hsacomo.org)  dr. Barsacchi Lucia |
|  |  | Azienda Ospedaliera Sant’Anna  Hospice Cure Palliative  Via Isonzo - 22066 Mariano Comense (Co) Responsabile dr. Longhi Carla [carla.longhi@hsacomo.org](mailto:carla.longhi@hsacomo.org)  dr. Nava Silvia; dr. Mariani Elisabetta |
|  | ***Marche*** | Ospedali Riuniti Umberto I Clinica Oncologica Via Conca, 71 Torrette - 60020 Ancona  *Responsabile dr. Tummarello Diego*  [d.tummarello@ao-umbertoprimo.marche.it](mailto:d.tummarello@ao-umbertoprimo.marche.it)  dr. Carbonari Giovanna |
|  |  | Azienda Ospedaliera Santa Croce ASL 3  Terapia del Dolore e Cure Palliative  Via Vittorio Veneto 2 - 61032 Fano (Pesaro Urbino)  *Responsabile dr. Fogliardi Alfredo*  [alfredo.fogliardi@asl3.marche.it](mailto:alfredo.fogliardi@asl3.marche.it)  Dr. Pozzi Rosangela |
|  |  | Ospedale Civile di Macerata  Divisione Oncologia Via Santa Lucia - 62100 Macerata Responsabile dr. Romagnoli Emanuela [emanuelaromagnoli@libero.it](mailto:eromagnoli@asl9.marche.it)  Dr. Valeri Michele |
|  |  | Osp Macerata ASL9 Marche  Terapia del Dolore  Via Santa Lucia - 62100 Macerata Responsabile dr. Nardi Luigi Filippo [lfnardi@asl9.marche.it](mailto:lfnardi@asl9.marche.it)  dr. Lombardello Marina |
|  | ***Piemonte*** | AO Santa Croce e Carle  Oncologia Medica Via M. Coppino, 26 - 12100 Cuneo  *Responsabile dr. Di Costanzo Gianna*  [dicostanzo.g@ospedale.cuneo.it](mailto:dicostanzo.g@ospedale.cuneo.it) |
|  |  | Ospedale Civile di Asti  Oncologia Medica Via Matteo Prandone 9 - 14100 Asti Responsabile dr. Testore Franco [francotestore@asl19.asti.it](mailto:francotestore@asl19.asti.it)  dr. Ceste Marco |
|  |  | Azienda Ospedaliera S.Lazzaro ASL 18 Oncologia Medica Via P. Belli, 26 - 12051 Alba (Cn)  *Responsabile dr. Porcile Gianfranco*  [gporcile@asl18.it](mailto:gporcile@asl18.it)  dr. Boe Maria Giovanna |
|  |  | ASL 15 Cuneo  Hospice di Busca  Cure Palliative  Piazza Regina Margherita 10 - 12022 Busca (Cn) Responsabile dr. La Ciura Pietro [curepalliative@asl15.sanitacn.it](mailto:curepalliative@asl15.sanitacn.it)  dr Nacca Roberto |
|  |  | ASL 12 Polo Oncologico di Biella  Oncologia Medica Via XX Settembre 25 -13900 Biella  *Responsabile dr. Clerico Mario*  [mario.clerico@asl12.piemonte.it](mailto:mario.clerico@asl12.piemonte.it)  dr. Loddo Carmen |
|  |  | Osp S.Giovanni Antica Sede  Oncologia Medica  Via Cavour, 31 - 10123 Torino  *Responsabile dr. Airoldi Mario*  [mairoldi@molinette.piemonte.it](mailto:mairoldi@molinette.piemonte.it)  dr. Berardo Roberto |
|  |  | Azienda Ospedaliera Molinette  Centro Universitario Ricerca Oncologica  Corso Bramante, 88 - 10126 Torino  *Responsabile dr. Ciuffreda Libero*  [lciuffreda@molinette.piemonte.it](mailto:lciuffreda@molinette.piemonte.it)  dr. Ottaviani Davide |
|  |  | Azienda Ospedaliera Molinette  Oncologia Medica  Corso Bramante, 88 - 10126 Torino  *Responsabile dr. Bertetto Oscar*  [obertetto@molinette.piemonte.it](mailto:obertetto@molinette.piemonte.it)  dr. Fissore Camilla |
|  |  | Azienda Ospedaliera Gradenigo  Oncologia  C.so Regina Margherita, 8 - 10153 Torino  *Responsabile dr.Comandone Alessandro*  [divisione.oncologia@h-gradenigo.it](mailto:divisione.oncologia@h-gradenigo.it)  dr. Dal Canton Orietta; dr. Garetto Alessandro |
|  |  | Osp Mauriziano Umberto I  Ginecologia Oncologica Largo Turati 62 - 10128 Torino  *Responsabile dr. Zola Paolo*  [pzola@mauriziano.it](mailto:pzola@mauriziano.it)  dr. Mazzola Simona |
|  |  | Azienda Ospedaliera San Luigi  Oncologia Medica Regione Gonzole, 10 - 10043 Orbassano (To)  *Responsabile dr. Dogliotti Luigi*  [luigi.dogliotti@unito.it](mailto:luigi.dogliotti@unito.it)  dr. Tucci Marcello |
|  |  | Azienda Ospedaliera ASL 9  Oncologia Medica P.zza Credenza 2 - 10015 Ivrea (To)  *Responsabile dr. Bretti Sergio*  [sergiobretti@hotmail.com](mailto:sergiobretti@hotmail.com)  dr. Bersano Giovanni |
|  |  | Osp. SS Antonio e Biagio  Oncologia  Via Venezia, 16 - 15100 Alessandria  *Responsabile dr. Bottero Guido*  [gbottero@ospedale.al.it](mailto:gbottero@ospedale.al.it)  dr. Blengio fulvia |
|  | ***Puglia*** | Ospedale Miulli  Medicina  Via M.Campagna 106 - 70021 Acquaviva Fonti Fg Responsabile dr. Lucarelli Giacomo [giacomo.lucarelli@tiscalinet.it](mailto:giacomo.lucarelli@tiscalinet.it)  dr. Nettis Giuseppe |
|  |  | Casa del Sollievo e Sofferenza, Osp S.G. Rotondo  U.O.Oncologia Via Cappuccini - 71013 S.Giovanni Rotondo (Ba)  *Responsabile dr.Morritti Maria*  [m.morritti@tiscali.it](mailto:m.morritti@tiscali.it)  dr. Di Maggio |
|  |  | IRCCS Oncologico  Oncologia Medica  Via Hahnemann, 209 - 70126 Bari  *Responsabile dr. Lorusso Vito*  [lorusso@email.it](mailto:lorusso@email.it)  dr. Calabrese Rita |
|  |  | IRCCS Oncologico  Terapia del Dolore e Cure Palliative Via Hahnemann, 209 - 70124 Bari  *Responsabile dr. Mattioli Vittorio*  [v.mattioli@oncologico.bari.it](mailto:v.mattioli@oncologico.bari.it)  dr. Aloè Ferruccio; dr. Montanaro Rosanna |
|  |  | Policlinico Consorziale di Bari  Medicina Interna e Oncologia Clinica Piazzale G. Cesare, 11 - 70100 Bari  *Responsabile dr.Dammacco Francesco*  [f.dammacco@dimo.uniba.it](mailto:f.dammacco@dimo.uniba.it)  dr. Iodice Giuseppe |
|  |  | Ospedale San Paolo ASL BA4  Oncologia  Via Capo Scardicchio, 10 - 70123 Bari Responsabile dr. Marzano Nicola [nicomrz@tiscali.it](mailto:nicomrz@tiscali.it)  dr. Altieri Michele |
|  |  | Ospedale Umberto I  Oncologia  Viale Regina Mergherita - 70022 Altamura (Ba) Responsabile dr. Vessia Giacomo [oncologia.altamura@virgilio.it](mailto:oncologia.altamura@virgilio.it)  dr. Abate Antonio |
|  |  | ASL BA1 Osp Civile Caduti in Guerra  DH Oncologico  Via G. Bovio, 81 - 70053 Canosa di Puglia (Ba)  *Responsabile dr.Cannone Michele*  [michelecannone@yahoo.it](mailto:michelecannone@yahoo.it)  dr. Carretta Alfonso |
|  |  | Azienda Ospedaliera di Molfetta ASL BA2  DH Oncologico  S.P. Molfetta Terlizzi - 70056 Molfetta (Ba)  *Responsabile dr. La Forgia Nicola*  Dr. De Palma Giovanni |
|  |  | Ospedale di Fasano - ASL BR1  Oncologia Medica  Via Naz.le dei Trulli, 95 - 72015 Fasano (Br)  *Responsabile dr. Ricciardi Giustina*  [giricci@katamail.com](mailto:giricci@katamail.com) |
|  |  | Azienda Ospedaliera Ferrari  Oncologia Medica  Via Circonvallazione - 73042 Casarano (Le)  *Responsabile dr. Schirinzi Attilio*  [legatumorilecce@tiscalinet.it](mailto:legatumorilecce@tiscalinet.it)  dr. Sapia Maria Assunta |
|  |  | Azienda Ospedaliera di Galatina  Oncologia Medica  Via Roma - 73013 Galatina (Le) Responsabile dr. Mancarella Sergio [mancarellasergio@libero.it](mailto:mancarellasergio@libero.it) |
|  |  | Ospedale di Nardò ASL LE1  Oncologia  Via XXV Luglio - 73048 Nardò (Le) Responsabile dr. Muci Dario [dario.muci@tin.it](mailto:dario.muci@tin.it) |
|  |  | Ospedale di Castellaneta  Oncologia Medica  Via del Mercato - 74011 Castellaneta (Ta) Responsabile dr. Rinaldi Antonio [toniorinaldi1@tin.it](mailto:toniorinaldi1@tin.it)  dr. Bruno Stefania |
|  | ***Sardegna*** | ASL 8 Azienda Ospedaliera Businco  Terapia Antalgica Via Edward Jenner - 09121 Cagliari  *Responsabile dr. Mameli Sergio*  [oncanestesia@tiscalinet.it](mailto:oncanestesia@tiscalinet.it)  dr. Pili Angela |
|  |  | Policlinico Universitario di Cagliari  Oncologia Medica II Strada Statale 55 - 09124 Monserrato (Ca)  *Responsabile dr. Massidda Bruno*  [masbru@pacs.unica.it](mailto:masbru@pacs.unica.it)  dr. Capra Daniela |
|  |  | Università di Sassari Clinica e Terapia Medica Viale San Pietro 43/b - 07100 Sassari  *Responsabile dr. Farris Antonio*  [afarris@uniss.it](mailto:afarris@uniss.it)  dr. Santeufemia Davide |
|  | ***Sicilia*** | A.O.Vittorio Emanuele Ospedale Santa Marta  Medicina del Dolore  Via Clementin 36 – 95124 Catania Responsabile dr. Chisari Sergio [s.chisari@tiscali.it](mailto:s.chisari@tiscali.it)  dr. Abbatista Carmelo |
|  |  | Centro Clinico Diagnostico Morgagni  Oncologia Medica  Via del Bosco, 105 - 95125 Catania Responsabile dr. Failla Giuseppe [oncomorg@hotmail.it](mailto:oncomorg@hotmail.it)  dr. Pappalardo Alessandro |
|  |  | Centro Catanese di Oncologia  Oncologia Medica via E. da Bormida, 64 - 95126 Catania Responsabile dr. Caruso Michele [michele.caruso@ccocatania.it](mailto:michele.caruso@ccocatania.it)  dr. Camillio Guido |
|  |  | Osp Garibaldi-Nesima  Oncologia Medica  Via Palermo, 632 - 95126 Catania Responsabile dr. Cordio Stefano [mmattina70@hotmail.com](mailto:mmattina70@hotmail.com)  dr. Mattina Marco |
|  |  | Osp Vittorio Emanuele II  Oncologia Medica  Via del Plebiscito, 632 - 95124 Catania  *Responsabile dr. Bordonaro Roberto*  [oncoct@hotmail.com](mailto:oncoct@hotmail.com)  dr. Buscarino Calogero |
|  |  | Ist Oncologico del Mediterraneo  Oncologia Medica 1 Via Penninazzo, 7 - 95029 Viagrande (Ct)  *Responsabile dr. Allegra Giuseppe*  [oncologiamedicaiom@virgilio.it](mailto:oncologiamedicaiom@virgilio.it)  dr. Vultaggio Giuseppe |
|  |  | Ist Oncologico del Mediterraneo  Dipartimento Oncologia  Via Penninazzo, 7 - 95029 Viagrande (Ct)  *Responsabile dr.Giuffrida Dario*  [dariogiuffrida@netscape.net](mailto:dariogiuffrida@netscape.net)  dr. Bianco Giusi |
|  |  | Policlinico G. Martino Oncologia Medica e Terapie Innovative Via Consolare Valeria - 98125 Messina  *Responsabile dr. Altavilla Giuseppe*  [galtavilla@unime.it](mailto:galtavilla@unime.it)  dr. Lupo Giuseppe |
|  |  | Policlinico G. Martino  Oncologia e Terapie Integrate  Via Consolare Valeria - 98125 Messina  *Responsabile dr. Adamo*  [adamovi@libero.it](mailto:adamovi@libero.it)  dr. Scandurra Giuseppina |
|  |  | Policlinico G. Martino  Oncologia Medica  Via Consolare Valeria - 98125 Messina  *Responsabile dr. La Torre Francesco*  [francesco.latorre@unime.it](mailto:Francesco.latorre@unime.it)  dr. Picone Giovanni |
|  |  | Casa di Cura Villa Salus  Oncologia Medica ed Ematologia  v.le Regina Margherita 12 – 98121 Messina  *Responsabile dr. Spadaro Pietro*  [dr.spadaro.pietro@virgilio.it](mailto:dr.spadaro.pietro@virgilio.it)  dr. Ingemi Maria Concetta |
|  |  | Azienda Ospedaliera San Vincenzo  Oncologia Medica Contrada Sirina - 98039 Taormina (Me)  *Responsabile dr. Ferraù Francesco; dr. Collina Paolo*  [omtaormina@tin.it](mailto:omtaormina@tin.it)  dr. Priolo Domenico; dr. Antonelli Giovanna |
|  |  | Centro Tumori “M. Ascoli”  Anestesia e Rianimazione  Via Parlavecchio, 143 - 90127 Palermo  *Responsabile dr. Di Stefano Marina*  [lumagi@libero.it](mailto:lumagi@libero.it)  dr. Scotto Silvia |
|  |  | Policlinico Universitario P. Giaccone  Oncologia Medica Via del Vespro, 129 - 90127 Palermo  *Responsabile dr. Gebbia Nicola*  [nicolagebbia@unipa.it](mailto:nicolagebbia@unipa.it)  dr. Fulfaro Fabio; dr. Rinaldi Gaetana |
|  |  | Clinica La Maddalena Oncologia Medica via San Lorenzo, 312 - 90146 Palermo  *Responsabile dr. Gebbia Vittorio*  [vittorio.gebbia@tin.it](mailto:vittorio.gebbia@tin.it)  dr. Grasso Valentina; dr. Agueli Roberta |
|  |  | Azienda Ospedaliera Ragusa  Oncologia Medica P.zza Ospedale Civile, 1 - 97100 Ragusa  *Responsabile dr. Iacono Carmelo*  [ciacono@aospedaliera.rg.it](mailto:ciacono@ospedaleragusa.it)  dr. Amendola Pasquale |
|  |  | Ospedale “Abele.Ajello”  Anestesia e Rianimazione  Via Salemi, 175 - 91026 Mazara del Vallo (Tp) Responsabile dr. Vaccaro Goffredo [g.vaccaro@tele2.it](mailto:g.vaccaro@tele2.it) |
|  | ***Toscana*** | Azienda Ospedaliera Careggi  Oncologia Medica Viale Morgagni, 85 - 50134 Firenze  *Responsabile dr. Di Costanzo Francesco*  [dicostanzofrancesco@tiscali.it](mailto:dicostanzofrancesco@tiscali.it)  dr. Vellucci Renato |
|  |  | ASL 6 Livorno  Oncologia Medica  Viale Alfieri, 36 - 57100 Livorno  Responsabile: dr. Falcone Alfredo  [m.andreuccetti@nord.usl6.toscana.it](mailto:m.andreuccetti@nord.usl6.toscana.it)  dr. Galli Luca; dr. Andreuccetti Michele |
|  |  | Azienda Ospedaliera Versilia ASL 12 Viareggio  Oncologia – Cure Palliative Via Aurelia, 335 - 55043 Camaiore (Lu)  *Responsabile dr. Buono Aurelio*  [d.amoroso@usl12.toscana.it](mailto:d.amoroso@usl12.toscana.it)  dr. Amoroso Domenico |
|  |  | Azienda Ospedaliera Universitaria Santa Chiara  Oncologia Medica Via Roma, 67 - 56100 Pisa  *Responsabile dr. Ricci Sergio*  [s.ricci@ao-pisa.toscana.it](mailto:s.ricci@mail.ao-pisa.toscana.it)  dr. Pastina Ilaria |
|  | ***Trentino Alto Adige*** | Ospedale S. Chiara  Oncologia Medica Largo Medaglie d'Oro - 38100 Trento Responsabile dr. Lucenti Antonio [antonio.lucenti@apss.tn.it](mailto:antonio.lucenti@apss.tn.it)  dr. Valduga Francesco |
|  | ***Umbria*** | Azienda Ospedaliera Santa Maria  Oncologia  Via Tristano di Joannuccio, 1 - 05100 Terni  *Responsabile dr. Buzzi Franco*  [buzzif@aospterni.it](mailto:buzzif@aospterni.it)  dr. Fumi Guglielmo |
|  |  | ASL 1  UO Oncologia  Località Chiocciolo  06012 Città Di Castello (PG)  *Responsabile* dr. Stefano Bravi  [stefano.bravi@asl1.umbria.it](mailto:stefano.bravi@asl1.umbria.it)  dr. Luigi Castori |
|  |  | Servizio Interdistrettuale di Cure Palliative - ASL 2  via Fuori Portauova  06081 Assisi (PG)  *Responsabile* dr. Barnabei Claudia  [oncologia@ausl2.umbria.it](mailto:oncologia@ausl2.umbria.it)  dr. Paoletti Federico |
|  | ***Veneto*** | Azienda Ospedaliera – Università di Padova Farmacologia ed Anestesiologia Via Giustiniani 1 - 35128 Padova  *Responsabile dr. Ceccherelli Francesco*  [istaneri@unipd.it](mailto:istaneri@unipd.it)  dr. Zampieri Silvia |
|  |  | Ospedale Busonera - USL 16  Oncologia Medica Via Gattamelata, 64 - 35139 Padova Responsabile dr. Cartei Giuseppe [giuscartei.oncol@ulss16.padova.it](mailto:giuscartei.oncol@ulss16.padova.it)  dr. Ceravolo Renato |
|  |  | Azienda ULSS 12  Oncologia Medica via Don F.Tosatto 147 - 30173 Venezia Mestre  *Responsabile dr. Paccagnella Adriano*  [adriano.paccagnella@ulss12.ve.it](mailto:adriano.paccagnella@ulss12.ve.it) |
|  |  | Casa di Cura Policlinico San Marco  Cure di Supporto Oncologico  Via Zanotto, 40 - 30173 Venezia Mestre  *Responsabile dr. Menegaldo Lorenzo*  [lorenzo.menegaldo@libero.it](mailto:lorenzo.menegaldo@libero.it)  dr. Poles Giovanni |
|  |  | ULSS 13 Mirano – Regione Veneto  Ginecologia e Oncologia  Via Mariutto 67 - 30035 Mirano (Ve)  *Responsabile dr. Maggino Tiziano*  [tiziano.maggino@ulss13mirano.ven.it](mailto:tiziano.maggino@ulss13mirano.ven.it)  dr. Sartori F. |
|  |  | Azienda Ospedaliera Calvi ASL 13  Oncologia ed Ematologia Oncologica  Via San Giorgio, 3 - 30033 Noale (Ve)  *Responsabile dr. Azzarello Giuseppe*  [azzarello.oncmed.oncnoale@inwind.it](mailto:azzarello@oncmed.oncnoale@inwind.it)  dr. Silvestri Barbara; dr. Marchioro Giovanni |
|  |  | Azienda Ospedaliera San Bortolo ASL 6 Vicenza  Terapia del Dolore e Cure Palliative Via Ridolfi, 11 - 36100 Vicenza  *Responsabile dr. Trentin Leonardo*  [leonardo.trentin@ulssvicenza.it](mailto:leonardo.trentin@ulssvicenza.it)  dr. Visentin Marco |
|  |  | ULSS 4 Alto Vicentino Ospedale di Thiene  Oncologia Medica  Via Rasa,9 - 36016 Thiene (Vi) Responsabile dr. Perin Alessandra [aperin@ulss4.veneto.it](mailto:aperin@ulss4.veneto.it) |
|  |  | Ospedale Civile Maggiore  Oncologia Medica  Piazza Stefani, 1 - 37126 Verona  *Responsabile dr. Cetto Gianluigi*  [gianluigi.cetto@univr.it](mailto:gianluigi.cetto@univr.it)  dr. Zuliani Serena |
